# Serum fibroblast growth factor 1 and its association with pancreatic beta cell function and insulin sensitivity in adults with glucose intolerance

**DOI:** 10.3389/fendo.2023.1198311

**Published:** 2023-05-22

**Authors:** Ji Yoon Kim, Jimi Choi, Yeongkeun Kwon, Sungsoo Park, Sin Gon Kim, Nam Hoon Kim

**Affiliations:** ^1^ Division of Endocrinology and Metabolism, Department of Internal Medicine, Korea University College of Medicine, Seoul, Republic of Korea; ^2^ Division of Endocrinology and Metabolism, Department of Medicine, Samsung Medical Center, Sungkyunkwan University School of Medicine, Seoul, Republic of Korea; ^3^ Center for Obesity and Metabolic Diseases, Korea University Anam Hospital, Seoul, Republic of Korea; ^4^ Division of Foregut Surgery, Korea University College of Medicine, Seoul, Republic of Korea

**Keywords:** beta cell function, fibroblast growth factor 1, glucose intolerance, insulin secretion, insulin sensitivity

## Abstract

**Background:**

Beneficial role of fibroblast growth factor 1 (FGF1) in the regulation of glucose metabolism and adipose tissue remodeling was suggested in rodents. This study aimed to investigate the association between serum FGF1 levels and metabolic parameters in adults with glucose intolerance.

**Methods:**

Serum FGF1 levels were examined using an enzyme-linked immunosorbent assay in 153 individuals with glucose intolerance. Associations between serum FGF1 levels and metabolic parameters, including body mass index (BMI), glycated hemoglobin (HbA1c), and 75 g oral glucose tolerance test-derived parameters, including insulinogenic index (IGI), Matsuda insulin sensitivity index (ISI), and disposition index (DI), were examined.

**Results:**

Serum FGF1 was detected in 35 individuals (22.9%), possibly due to the autocrine/paracrine nature of the peptide. IGI and DI levels were significantly lower in individuals with higher FGF1 levels than in those with lower FGF1 levels or undetectable FGF1 (p=0.006 and 0.005 for IGI and DI, respectively, after adjustment for age, sex, and BMI). Univariable and multivariable analyses using the Tobit regression model also revealed a negative association between FGF1 levels and IGI and DI. The regression coefficients per 1-SD of log-transformed IGI and DI were −0.461 (p=0.013) and −0.467 (p=0.012), respectively, after adjustment for age, sex, and BMI. In contrast, serum FGF1 levels were not significantly associated with ISI, BMI, or HbA1c.

**Conclusions:**

The serum concentration of FGF1 was significantly elevated in individuals with low insulin secretion, suggesting a possible interaction between FGF1 and beta cell function in humans.

## Introduction

Fibroblast growth factor 1 (FGF1) is an autocrine/paracrine peptide that participates in adipose tissue remodeling and regulates glucose metabolism (1). FGF1 was induced in adipose tissue by a high-fat diet, and FGF1-lacking mice developed an aggressive diabetic phenotype with aberrant adipose expansion, suggesting a role of FGF1 in adipose remodeling (2). A single intracerebroventricular injection of FGF1 induced sustained diabetes remission for 18 weeks in mouse models of type 2 diabetes (3). The exact mechanism of the glucose-lowering effect of central FGF1 administration is not fully understood; hypothalamic neurons and glial cells, such as tanycytes and astrocytes, have been suggested as targets of the central action of FGF1 (4). Peripheral injection of FGF1 also reverses hyperglycemia partly by suppressing adipose lipolysis and reducing hepatic glucose production (5). Evidence suggests that FGF1 also regulates pancreatic beta cell function (6–8). Peripheral injection of FGF1 increases beta cell density and ex vivo insulin secretion in diabetic mouse islets (6).

However, the metabolic effects of FGF1, including insulin secretion and sensitivity, have not been well elucidated in humans. This study aimed to investigate the association between serum FGF1 levels and metabolic parameters in adults with glucose intolerance.

## Materials and methods

This study included participants from two separate cohorts from November 2016 to January 2022. The first cohort comprised participants aged ≥19 years who were diagnosed with glucose intolerance at a single tertiary hospital, Korea University Anam Hospital. Glucose intolerance was defined as fasting plasma glucose ≥100 mg/dL or 2-h glucose ≥140 mg/dL on the 75 g oral glucose tolerance test (OGTT) or HbA1c ≥5.7% (39 mmol/mol). The second cohort comprised adults with obesity, with a BMI ≥25 kg/m^2^, who were planning to undergo bariatric surgery at four tertiary medical centers in South Korea. The participants in both cohorts underwent blood tests after overnight fasting. Serum samples were stored at −70°C before FGF1 analysis. Serum FGF1 levels were examined using an enzyme-linked immunosorbent assay (catalog no. DFA00B; R&D Systems, Inc., Minneapolis, MN USA). Samples were analyzed in duplicates.

All participants underwent a 75 g OGTT at enrollment. Serum glucose and insulin levels were measured at fasting (0), 30, and 120 min after 75 g glucose loading. Insulin secretion was estimated using the insulinogenic index (IGI), which was calculated as (insulin_30 min_ – insulin_0 min_ [μIU/mL])/(glucose_30 min_ – glucose_0 min_ [mg/dL]) (9). Insulin sensitivity was measured using the Matsuda insulin sensitivity index (ISI), calculated as 10000/(fasting glucose [mg/dL] × fasting insulin [μIU/mL] × mean glucose [mg/dL] × mean insulin [μIU/mL])^1/2^ (10). The disposition index (DI), reflecting beta cell function adjusted for insulin sensitivity, was calculated as IGI × ISI (11).

Individuals were divided into three groups—undetected FGF1, low FGF1, and high FGF1—and their clinical characteristics were compared. We also used a Tobit regression model with a lower bound of half of the observed minimum value to assess the association between FGF1 and metabolic parameters, since there was censoring below 0.056 pg/mL at the observed FGF1 value. Information on age, sex, hypertension, dyslipidemia, and cardiovascular disease was collected using a questionnaire. BMI, waist circumference, systolic blood pressure, total cholesterol, HbA1c, fasting glucose, alanine aminotransferase, and creatinine levels were measured. Variables with right-skewed distributions were transformed logarithmically. The Tobit regression model included log-transformed FGF1 as a dependent variable and each metabolic parameter, log-transformed IGI, ISI, and DI as independent variables. The model was adjusted for age, sex, and BMI. The results are presented as the regression coefficient per 1-SD of the independent variable and the standard error of the coefficient. Additionally, we evaluated the difference in mean serum FGF1 levels according to the quartile of each variable, adjusted for age and sex. All statistical analyses were performed using the SAS software (version 9.4; SAS Institute Inc., Cary, NC, USA). Statistical significance was set at a two-sided p-value of <0.05.

This study was approved by the Institutional Review Board of Korea University Anam Hospital (IRB number 2017AN0050). Written informed consent was obtained from all participants.

## Results

The characteristics of the 153 participants are presented in **Table 1**. The mean age of the patients was 50 years, and 46.4% were female. The mean BMI was 31.7 kg/m^2^ and the mean HbA1c level was 54 mmol/mol (7.1%). Type 2 diabetes was present in 88.2% of the participants, and 55.6% of the participants received anti-diabetic agents.

**Table 1:** Baseline characteristics of study participants.

	Total (N=153)	
Age (years)^a^	50.3	(12.9)
Female, n (%)^b^	71	(46.4)
Diabetes, n (%)^b^	135	(88.2)
Anti-diabetic agents use, n (%)^b^	84	(55.6)
Metformin, n (%)^b^	77	(51.0)
Sulfonylurea, n (%)^b^	21	(13.9)
Glinide, n (%)^b^	1	(0.7)
GLP-1R agonist, n (%)^b^	3	(2.0)
DPP-4 inhibitor, n (%)^b^	25	(16.6)
Thiazolidinedione, n (%)^b^	8	(5.3)
SGLT2 inhibitor, n (%)^b^	35	(23.2)
Insulin, n (%)^b^	12	(7.9)
Hypertension, n (%)^b^	110	(72.4)
Dyslipidemia, n (%)^b^	123	(80.9)
Body mass index (kg/m^2^)^a^	31.7	(8.3)
Waist circumference (cm)^a^	102.7	(22.3)
Systolic blood pressure (mmHg)^a^	133.7	(13.2)
Total cholesterol (mg/dL)^a^	179.8	(51.7)
Alanine aminotransferase (IU/mL)^a^	41.7	(43.2)
Creatinine (mg/dL)^a^	0.80	(0.18)
HbA1c (mmol/mol)^a^	54.1	(17.7)
HbA1c (%)^a^	7.1	(1.6)
Fasting glucose (mg/dL)^a^	140.6	(43.9)
Ln(IGI)^a^	-1.52	(1.24)
Ln(ISI)^a^	-1.39	(0.64)
Ln(DI)^a^	-2.95	(1.20)

Variables with ^a^ are presented as the means (standard deviation), while variables with ^b^ are presented as the numbers (%).

DI, disposition index; DPP-4, dipeptidyl peptidase-4; GLP-1 R, glucagon-like peptide-1 receptor; IGI, insulinogenic index; ISI, Matsuda insulin sensitivity index; SGLT2, sodium glucose cotransporter 2.

Serum FGF1 was detected in 35 participants (22.9%), and their mean serum FGF1 level was 5.07 pg/mL. Individuals with detectable FGF1 were younger and had a higher BMI than those without. While HbA1c, IGI, and ISI were not significantly different between the groups, DI was significantly lower in individuals with detectable FGF1 than in others (Ln(DI); -3.32 *vs*. -2.84, p=0.043) (**Table 2**).

**Table 2:** Clinical characteristics of individuals with undetectable and detectable serum FGF1 levels.

	Individuals with undetectable FGF1 (N=118)		Individuals with detectable FGF1 (N=35)		*P*
Age (years)^a^	51.5	(12.5)	46.3	(13.4)	0.033
Female, n (%)^b^	53	(44.9)	18	(51.4)	0.497
Diabetes, n (%)^b^	104	(88.1)	31	(88.6)	0.944
Anti-diabetic agents use, n (%)^b^	66	(56.4)	18	(52.9)	0.720
Metformin, n (%)^b^	60	(51.3)	17	(50.0)	0.895
Sulfonylurea, n (%)^b^	14	(12.0)	7	(20.6)	0.258
Glinide, n (%)^b^	0	(0.0)	1	(2.9)	0.225
GLP-1R agonist, n (%)^b^	2	(1.7)	1	(2.9)	0.538
DPP-4 inhibitor, n (%)^b^	20	(17.1)	5	(14.7)	0.742
Thiazolidinedione, n (%)^b^	6	(5.1)	2	(5.9)	>0.999
SGLT2 inhibitor, n (%)^b^	25	(21.4)	10	(29.4)	0.328
Insulin, n (%)^b^	7	(6.0)	5	(14.7)	0.143
Hypertension, n (%)^b^	85	(72.0)	25	(73.5)	0.864
Dyslipidemia, n (%)^b^	97	(82.2)	26	(76.5)	0.454
Body mass index (kg/m^2^)^a^	30.8	(7.4)	34.9	(10.4)	0.034
Waist circumference (cm)^a^	100.2	(20.0)	111.3	(27.4)	0.032
Systolic blood pressure (mmHg)^a^	132.7	(12.8)	137.1	(13.9)	0.082
Total cholesterol (mg/dL)^a^	179.7	(51.2)	180.2	(54.1)	0.956
Alanine aminotransferase (IU/mL)^a^	40.5	(45.1)	45.9	(36.0)	0.514
Creatinine (mg/dL)^a^	0.81	(0.19)	0.74	(0.16)	0.047
HbA1c (mmol/mol)^a^	53.5	(17.2)	56.2	(19.6)	0.446
HbA1c (%)^a^	7.1	(1.6)	7.3	(1.8)	0.446
Fasting glucose (mg/dL)^a^	139.3	(44.5)	145.0	(42.1)	0.506
Ln(IGI)^a^	-1.44	(1.20)	-1.81	(1.34)	0.132
Ln(ISI)^a^	-1.35	(0.66)	-1.51	(0.56)	0.219
Ln(DI)^a^	-2.84	(1.12)	-3.32	(1.39)	0.043

Variables with ^a^ are presented as the means (standard deviation) and compared using Student’s t-test. Variables with ^b^ are presented as the numbers (%) and compared using the chi-square test or Fisher's exact test.

DI, disposition index; DPP-4, dipeptidyl peptidase-4; GLP-1 R, glucagon-like peptide-1 receptor; IGI, insulinogenic index; ISI, Matsuda insulin sensitivity index; SGLT2, sodium glucose cotransporter 2.

**Figure 1:**
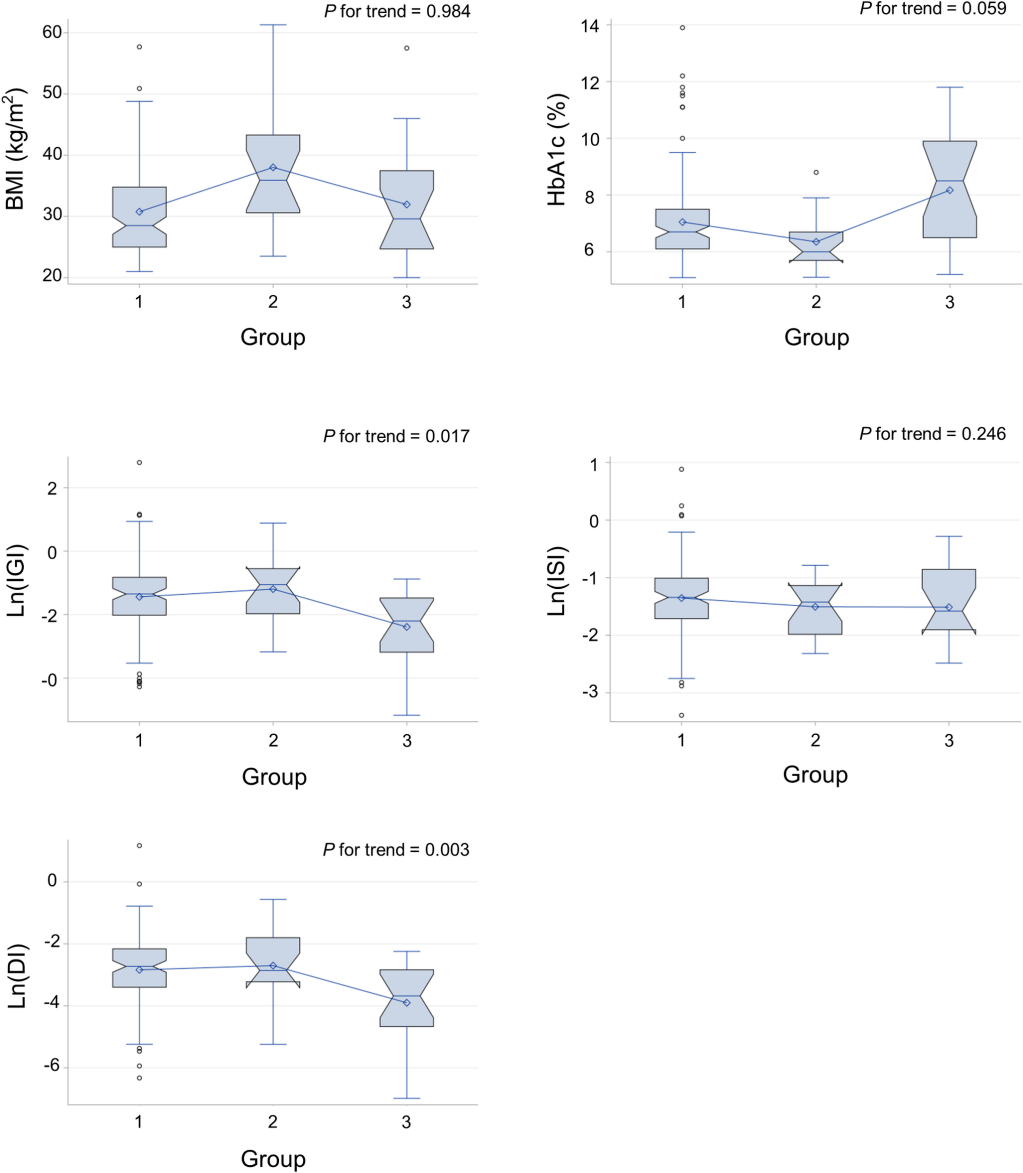
Metabolic parameters of individuals with undetectable (group 1), low (group 2), and high (group 3) serum FGF1 levels. DI, disposition index; IGI, insulinogenic index; ISI, Matsuda insulin sensitivity index.

Comparing the metabolic parameters between the three groups (undetectable, low, and high FGF1) revealed a negative correlation between Ln(IGI) and Ln(DI) and FGF1 levels (p=0.006 and 0.005, respectively, after adjustment for age, sex, and BMI). However, no significant association with BMI and HbA1c was found (**Table 3**; **Figure 1**).

**Table 3:** Metabolic parameters of individuals with undetectable (group 1), low (group 2), and high (group 3) serum FGF1 levels.

	Group 1		Group 2		Group 3		*p* for trend		
	(N=118)		(N=17)		(N=18)		Unadjusted	Age and sex adjusted	Age, sex, and BMI adjusted
BMI (kg/m^2^)	30.8	(7.4)	38.0	(10.4)	32.0	(9.7)	0.984	0.375	
HbA1c (%)	7.1	(1.6)	6.4	(1.0)	8.2	(1.9)	0.059	0.062	0.056
Ln(IGI)	-1.4	(1.2)	-1.2	(1.1)	-2.4	(1.3)	0.017	0.008	0.006
Ln(ISI)	-1.4	(0.7)	-1.5	(0.5)	-1.5	(0.6)	0.246	0.555	0.770
Ln(DI)	-2.8	(1.1)	-2.7	(1.2)	-3.9	(1.3)	0.003	0.004	0.005

Data is presented as the means (standard deviation). Individuals with detectable FGF1 were divided into group 2 (lower half) and 3 (upper half) by the median of serum FGF1 levels.

DI, disposition index; IGI, insulinogenic index; ISI, Matsuda insulin sensitivity index.

In univariable analysis using the Tobit regression model, serum FGF1 levels were negatively associated with IGI (β=−0.387, p=0.039) and DI (β=−0.487, p=0.010) (**Table 4**). No significant association was found between serum FGF1 levels and ISI (β=−0.217, p=0.251), BMI (β=0.313, p=0.089), or HbA1c (β=0.291, p=0.117). IGI and DI remained significant after adjusting for age and sex (β=−0.438, p=0.019; β=−0.475, p=0.011, respectively) and after further adjusting for BMI (β=−0.461, p=0.013; β=−0.467, p=0.012, respectively).

**Table 4:** Univariable and multivariable analysis of the association between serum FGF1 levels and metabolic parameters using a Tobit regression model.

	Unadjusted			Age and sex adjusted			Age, sex, and BMI adjusted		
Outcome: Ln(FGF1)	β	SE	*p*	β	SE	*p*	β	SE	*p*
BMI per 1 SD	0.313	0.184	0.089	0.180	0.212	0.395			
HbA1c per 1 SD	0.291	0.186	0.117	0.289	0.185	0.119	0.298	0.185	0.107
Ln(IGI) per 1 SD	–0.387	0.188	0.039	–0.438	0.186	0.019	–0.461	0.186	0.013
Ln(ISI) per 1 SD	–0.217	0.189	0.251	–0.106	0.194	0.586	–0.051	0.202	0.801
Ln(DI) per 1 SD	–0.487	0.189	0.010	–0.475	0.187	0.011	–0.467	0.187	0.012

A Tobit regression model with a lower bound of half the observed minimum value of FGF1 was used. Results are presented as the regression coefficient per 1 standard deviation of the independent variable (β) and standard error (SE) of the coefficient.

DI, disposition index; IGI, insulinogenic index; ISI, Matsuda insulin sensitivity index.

**Figure 2:**
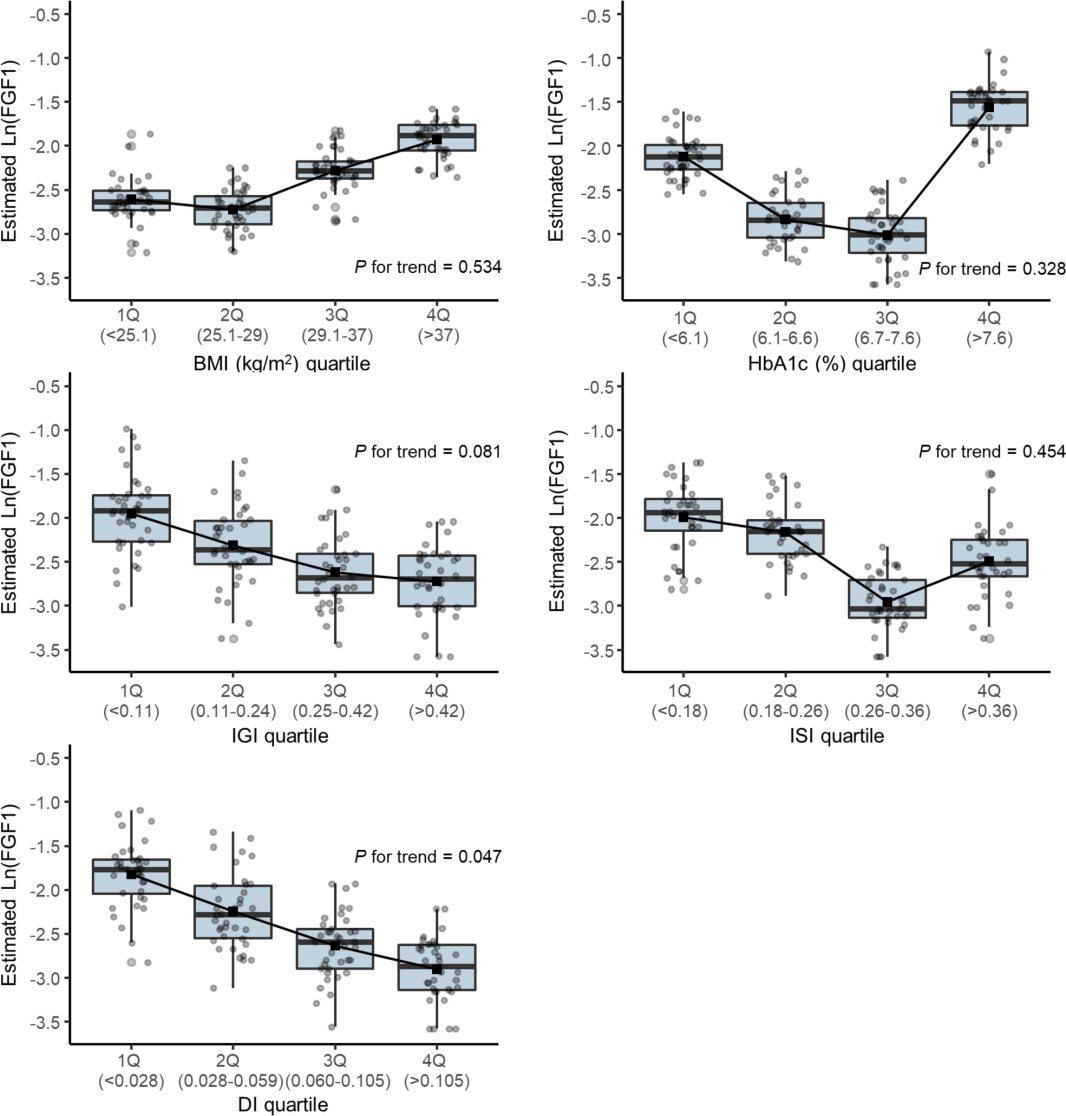
Box plot of the estimated log-transformed serum FGF1 level according to the quartile of each parameter using a Tobit regression model adjusted by age and sex. DI, disposition index; IGI, insulinogenic index; ISI, Matsuda insulin sensitivity index.

**Figure 2** shows the box plot of the estimated log-transformed FGF1 levels according to the quartile of each parameter using the Tobit regression model adjusted for age and sex. The mean FGF1 concentrations were significantly lower in the higher quartiles of DI (p for trend=0.047).

## Discussion

This study found that serum FGF1 levels were significantly higher in individuals with low insulin secretion. Serum FGF1 had a negative correlation with IGI and DI in both the unadjusted and adjusted models, suggesting a possible interaction between FGF1 and insulin secretion or beta cell function in humans. To the best of our knowledge, this is the first human study to address the association between circulating FGF1 levels and insulin indices derived from 75 g OGTT.

It has been demonstrated that FGF1 partially ameliorates diabetes-induced beta cell dysfunction by improving insulin secretion at the pancreatic islet level. Central and peripheral FGF1 administration increased ex vivo islet insulin secretion in diabetic mice, and peripheral FGF1 increased islet beta cell density (6). In zebrafish, overnutrition induced compensatory beta cell differentiation *via* FGF1 signaling. Inactivation of *fgf1* abolished compensatory beta cell differentiation. Expression of FGF1 in beta cells of *fgf1^-/-^* animals rescued the compensatory response (7). In another study, central FGF1 injection delayed the onset of progressive beta cell loss in a rat model of diabetes (8). Taken together, FGF1 increases insulin secretion and preserves beta cell function in multiple ways, partly contributing to its prolonged glucose-lowering effect in rodents. In our study, the serum FGF1 concentration was higher in individuals with impaired beta cell function. This finding implies that FGF1 has an association with beta cell function in humans. Our findings are consistent with previous findings that the HOMA-B was lower in participants with higher FGF1 levels among patients newly diagnosed with type 2 diabetes and healthy controls (12). We postulate that serum FGF1 levels may increase in individuals with low insulin secretion to compensate for beta cell dysfunction. However, our cross-sectional study design could not determine causal relationships. 

FGF1 is an autocrine or paracrine hormone. FGF1 has a high affinity for heparan sulfate proteoglycans, which are components of the extracellular matrix that modulate FGF/FGF receptor signaling. This high affinity for heparan sulfate proteoglycan hinders the release of FGFs from the originating tissues into circulation (5). In our study, FGF was detected in 22.9% of the participants. To validate the assay, we used the samples from multi-centers, and the experiment was done by two different researchers. All the samples were analyzed in duplicates, and the coefficient of variation of samples was less than 15%. The low detection rate of circulating FGF1 in our study supports that FGF1 is an autocrine/paracrine hormone rather than an endocrine hormone. This is in line with previous studies showing that circulating FGF1 was detected in 5 out of 31 subjects (13) and serum FGF1 was not detected in healthy humans (14). Nonetheless, the low detection rate of serum FGF1 was a limitation of this study. Therefore, we used Tobit regression analysis to handle censored data. In contrast, circulating FGF1 was well detected in some previous studies (12, 15, 16), which may be due to the different assay methods and detection ranges, and different study populations.

Previous studies showed conflicting results on the association between FGF1 and BMI or adiposity measures. Wang et al. reported positive associations between BMI and FGF levels (12, 15) whereas Zhu et al. showed negative associations between BMI and FGF1 levels (16). Mejhert et al. reported that FGF1 expression in subcutaneous white adipose tissue was elevated in obese people, however, this did not contribute to the circulating levels (13). A preclinical study showed an insulin-sensitizing effect of FGF1 when it was pharmacologically given, suggesting a possible interaction between FGF1 and insulin sensitivity (4, 17). However, our study did not show any significant association between serum FGF1 levels and BMI or insulin sensitivity. The discrepancy between local expression, circulating level, and pharmacologic action of FGF1 suggests its complex and undiscovered nature. Further studies are required to validate the metabolic actions of FGF1.

In conclusion, serum FGF1 levels are significantly higher in individuals with lower insulin secretion. This suggests a possible interaction between FGF1 and insulin secretion in humans. Future studies are needed to reveal the exact mechanism by which FGF1 modulates beta cell function in humans.

## Data availability statement

The original contributions presented in the study are included in the article/supplementary material. Further inquiries can be directed to the corresponding author.

## Ethics statement

The studies involving human participants were reviewed and approved by Institutional Review Board of Korea University Anam Hospital. The patients/participants provided their written informed consent to participate in this study.

## Author contributions

JK: Conceptualization, Investigation, Writing – original draft, Writing – review and editing. JC: Formal analysis, Methodology. YK: Investigation. SP: Resources. SK: Resources. NK: Conceptualization, Funding acquisition, Project administration, Supervision, Writing – review and editing. All authors contributed to the article and approved the submitted version.
